# Imprinted Polymeric Beads-Based Screen-Printed Potentiometric Platforms Modified with Multi-Walled Carbon Nanotubes (MWCNTs) for Selective Recognition of Fluoxetine

**DOI:** 10.3390/nano10030572

**Published:** 2020-03-21

**Authors:** Saad S.M. Hassan, Ayman H. Kamel, Abd El-Galil E. Amr, Heba M. Hashem, E.M. Abdel Bary

**Affiliations:** 1 Chemistry Department, Faculty of Science, Ain Shams University, Abbasia, Cairo 11566, Egypt; hebahashem426@yahoo.com; 2 Pharmaceutical Chemistry Department, Drug Exploration & Development Chair (DEDC), College of Pharmacy, King Saud University, Riyadh 11451, Saudi Arabia; 3 Applied Organic Chemistry Department, National Research Center, Dokki, Giza 12622, Egypt; 4 Chemistry Department, Faculty of Science, Mansoura University, Mansoura 35516, Egypt; ebary301@yahoo.com

**Keywords:** Solid-contact ISEs, multi-walled carbon nanotubes (MWCNTs), fluoxetine, screen-printed electrodes, method validation

## Abstract

Herein, we present a new validated potentiometric method for fluoxetine (FLX) drug monitoring. The method is based on the integration of molecular imprinting polymer (MIP) beads as sensory elements with modified screen-printed solid contact ion-selective electrodes (ISEs). A multi-walled carbon nanotube (MWCNT) was used as a nanomaterial for the ion-to-electron transduction process. The prepared MIP beads depend on the use of acrylamide (AAm) and ethylene glycol dimethacrylic acid (EGDMA) as a functional monomer and cross-linker, respectively. The sensor revealed a stable response with a Nernstian slope of 58.9 ± 0.2 mV/decade and a detection limit of 2.1 × 10^−6^ mol/L in 10 mmol/L acetate buffer of pH 4.5. The presented miniaturized sensors revealed good selectivity towards FLX over many organic and inorganic cations, as well as some additives encountered in the pharmaceutical preparations. Repeatability, reproducibility and stability have been studied to evaluate the analytical features of the presented sensors. These sensors were successfully applied for FLX assessment in different pharmaceutical formulations collected from the Egyptian local market. The obtained results agreed well with the acceptable recovery percentage and were better than those obtained by other previously reported routine methods.

## 1. Introduction

Fluoxetine (FLX) is one of the five drugs inserted under the selective serotonin re-uptake inhibitors (SSRIs) category that is used throughout the world as an anti-depressant drug. Depression diagnosis can be expressed as a mental health illness and disability. According to the world federation for mental health, depression is occurring as a reason for different problems that affect the behavior of humans. It can cause economic problems, increase the rate of unemployment and it is a main reason for family disturbance [1]. It can also cause occupational stress when the worker is facing work demands and is not matched with its requirements [2]. Fluoxetine hydrochloride was approved by the US Food and Drug Administration (FDA) organization since 1987 for depression treatment [3]. It is administered by the oral route and possesses a unique effect on the obsessive-compulsive disorder [4,5]. 

There are different methods of FLX determination in pharmaceutical forms and biological fluids reported in the literature. Among these methods are spectrophotometry [5,6,7,8], capillary electrophoresis [9,10,11], high-performance liquid chromatography (HPLC) [12,13], gas chromatography-mass spectrometry technique [14,15], fluorimetry [16], voltammetry [17,18,19,20] and potentiometry [21,22,23]. 

Unlike the conventional liquid-contact ISEs, solid-contact ISEs which eliminate the internal solution, are easily miniaturized and have been recognized as the next generation of ISEs. It is well known that the electrode substrates for these electrodes are usually made from expensive materials such as glassy carbon, gold or platinum. This reveals serious limits to their wide use, especially in less developed countries. For cost-effectiveness, fast, accessible and precise analysis, simple instrumentation, and incorporated functionality, screen-printed ISEs have been chosen for flexible, reliable and low-cost platforms for potentiometric analytical devices [24,25,26]. In addition, this type of electrode offers a practical viable method without sample pretreatment, prolonged analysis time and sophisticated experimental establishment.

Molecularly imprinted polymers (MIPs) possess pre-defined specific recognition cavities designed for the target analyte. They are stable to extremes of pH, organic solvents and temperature, which provides for more flexibility in the development of analytical and bioanalytical methods [27,28,29]. MIPs have a valuable impact on the enhancement of ion-selective electrodes, in which the encapsulated molecules, attached via their high affinity three-dimensional cavities, act as tailor-made highly specific receptor sites for the desired molecule [30,31,32,33,34,35,36]. In addition, the developed membrane potential in ISEs does not require the extraction of the template from the molecular imprinting skeleton. There are also no size restrictions on the template compound because species do not have to diffuse through the membrane.

Herein, a new, simple and cost-effective method for FLX determination using a ceramic screen-printed planar electrode is presented. Artificial receptors for FLX based on template imprinted polymers were synthesized using thermal precipitation polymerization and acrylamide (AAm) as an appropriate monomer. The properties of the proposed sensor can be studied via different features, which reflect the high sensitivity, selectivity with low potential drifts, stability and more applicability of investigated sensors in pharmaceutical preparations.

## 2. Materials and Methods 

### 2.1. Reagents and Chemicals

For MIPs synthesis and membrane fabrication, all chemicals were obtained from Sigma Aldrich (St. Louis, Missouri, MO, USA) such as high molecular weight poly (vinyl chloride) (PVC), dioctyl phthalate (DOP), 2-nitrophenyl octyl ether (o-NPOE), bis (2-Ethylhexyl) sebacate (DOS), sodium tetraphenylborate (TPB), acrylamide (AAm), benzoyl peroxide (BPO) and ethylene glycol dimethacrylate acid (EGDMA 98%). Tetrahydrofuran (THF) and acetonitrile were obtained from Fluka AG (Buchs, Switzerland). Tetrahydrofuran (THF) was freshly distilled prior to use. In addition, MWCNTs were purchased from (EPRI, Cairo, Egypt). Fluoxetine.HCl pure drug was obtained from Pharaonia Pharmaceuticals (Alexandria, Egypt). Fluoxetine capsules were obtained from pharmacies in Egypt, which were represented with different commercial names such as Prozac (Lilly, France), Philozac (Amoun, Egypt), Flutin (Eipico, Egypt) and Depreban (Amirya, Egypt). All these commercial names contained 20 mg FLX per capsule.

A definite weight of pure FLX·HCl drug was dissolved in 100 mL double distilled water to prepare a stock solution of 1.0 × 10^−2^ mol/L. Also, 10 mmol/L acetate buffer solution of pH 4.5 was prepared for calibration measurements. The working solutions (1.0 × 10^−2^–1.0 × 10^−7^ mol/L) were prepared with accurate dilutions and stored in brown bottles in the refrigerator.

### 2.2. Apparatus

An attenuated total reflection (ATR) Fourier transform spectrometer (Thermo-Fisher Scientific iS10, Austin, TX, USA) and a scanning electron microscope (SEM) (JEOL JSM 6510lV, Osaka, Japan) were used for characterization of MIP particles. The binding analysis was investigated by using a Shimadzu UV/VIS spectrophotometer (Shimadzu UV-1601 PC, Osaka, Japan) for absorbance measurements. A bench pH/mV meter (Jenway™ 3510) was used for all potential measurements as a combination of drop-casted FLX membrane on the screen-printed and Jenway™Ag/AgCl double junction reference electrode filled in the outer compartment with 1 M CH_3_COOLi. For necessary pH measurements, a Jenway™ 3505 combined glass pH electrode was used. Also, the reference method was applied by using high-performance liquid chromatography (HPLC) coupled with a UV/VIS detector (Series 200 Pump, Perkin Elmer, Waltham, MA, USA).

### 2.3. Synthesis of MIPs

**Figure 1:**
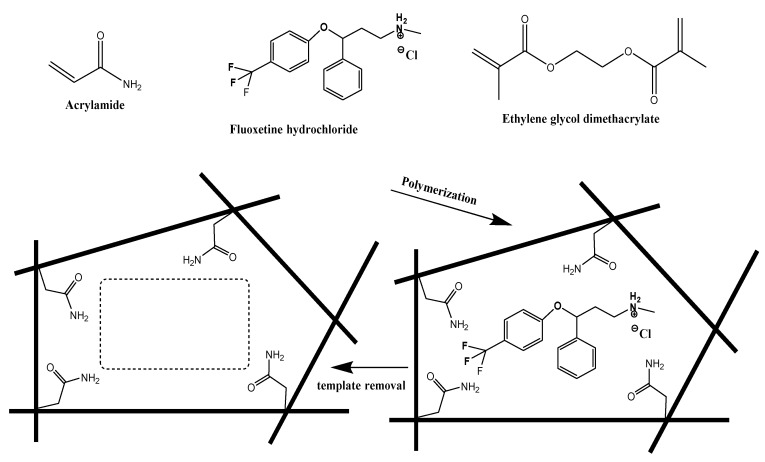
Schematic representation of noncovalent imprinting.

Host-tailored three-dimensional polymeric particles (MIPs) were synthesized with precipitation polymerization. An amount of 3.0 mmol of cross-linked AAm monomer with 3.0 mmol of EGDMA was mixed with 1.0 mmol of FLX as a template. The polymerization process was initiated by adding 80 mg of BPO into a cocktail that was dissolved in 15 mL of acetonitrile in (20 mL) a sealed tube. To complete the polymerization, the N_2_ stream was diffused into the cocktail solution for 5 min, followed by good sealing of the tube. Also, the tube was maintained in paraffin wax at 70 °C for 20 h. All previous steps were repeated for non-imprinted polymer (NIP) synthesis without the addition of the FLX template. The resulting powders were washed after drying with absolute ethanol several times in soxhlet for 48 h. MIP and NIP were left until complete dryness at room temperature before using them. A scheme of the stepwise fabrication process of the biomimetic receptor is presented in Figure 1.

### 2.4. Fabrication of the Sensors and EMF Measurements

The screen-printed electrode (SPE) is fabricated as a planar chip of 0.1 mm thickness and 35 mm length containing two sensing orifices of 2 mm width. These orifices were made from carbon ink and printed on an alumina substrate to be the area of the membranes’ drop-castings. Different membranes were prepared with 68.0 mg of PVC powder, 2.0 mg of additive (TPB) and 12.0 mg of MIP. The previous membranes contained 118.0 mg of different plasticizers such as DOP, *o*-NPOE or DOS and 3.0 mL of THF was used to dissolute all previous components. The screen-printed chip was modified with drop-casting of 10 µL of MWCNTs (0.1 g/25 mL THF) on sensing orifices onto which 10 µL of ionophore containing membranes was drop-casted and was left to dry for 5 min.

Then, these sensors were soaked in 1.0 × 10^−2^ mol/L FLX solution for 2 h before using. The same solution was also used for sensors storage. All potential measurements were applied after potential stabilization ± 2 mV and the results were plotted, to display the resulting EMF values versus logarithm [FLX] concentration obtained.

### 2.5. Analytical Applications

FLX was administered via the oral route, which was represented commercially as Prozac, Philozac, Flutin and Depreban capsules containing 20 mg/capsule. A stock of FLX was prepared by mixing 20 capsules’ contents (20 mg per one capsule), weighing and calculating the mean weight of the active ingredient in one capsule. An accurately weighed amount of the powder equivalent to 3.4 g of FLX was dissolved in 100 mL dist-H_2_O to obtain 10^−1^ mol/L FLX stock. The previous contents were sonicated until complete dissolution for 45 min and the resultant solution was filtered. Different concentrations were prepared from dilution of the supernatant to obtain 10^−2^ to 10^−4^ mol/L. The calibration curve was established between the potential readings versus log [FLX] and compared with the similar curve of the pure drug under the same conditions.

## 3. Results and Discussion

### 3.1. Characterization of the MIP Particles

**Figure 2:**
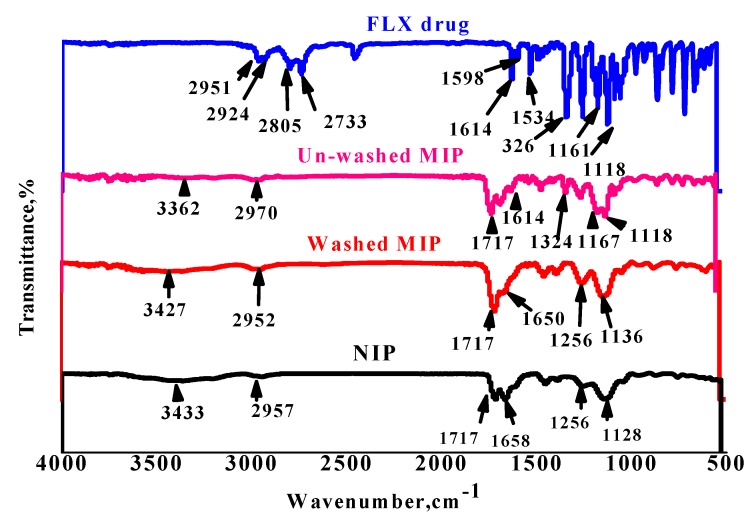
FT-IR spectra of fluvoxamine (FLX), FLV/MIP, washed molecular imprinting polymer (MIP) and non-imprinted polymer (NIP) beads.

Figure 2 shows the FT-IR spectra of the FLX drug, unwashed and washed MIP, in addition to the NIP beads. First, strong peaks of FLX that appeared at 2951 and 2924 cm^−1^ are attributed to the asymmetric C–H stretches and the three strong peaks at 2805, 2733 and 2450 cm^−1^ refer to NH_2_^+^ stretches. All peaks that appeared at 1614, 1598 and 1534 cm^−1^ exhibited from phenyl ring vibrations and C=C stretching. C–F stretching is assigned at 1326 cm^−1^. Strong and sharp peaks at 1161 and 1118 cm^−1^ attributed to C–N stretches of a secondary amine and –C–O in aryl ether. In the unwashed MIP spectrum, it can be shown that the clear peaks at 3362 and 1167 cm^−1^ refer to N–H stretching and C–N of the secondary amine group that present in FLX, respectively. Medium peaks at 1614 and 1324 cm^−1^ attributed to phenyl ring vibrations and C–F stretches, which appeared in FLX spectra, confirm the progress of the imprinting process between FLX and cross-linked AAm monomer. The strong and sharp peak of cross-linked AAm monomer in MIPs and NIPs that appeared at 1717 cm^−1^ corresponds to –C=O and all peaks from 1256 to 1128 cm^−1^ corresponds to –C–O stretching of the polymeric backbone. In washed MIP and NIP, it is clear that there is a complete disappearance of assignable peaks at 1614 and 1324 cm^−1^ that refer to phenyl ring vibrations and C–F stretches in FLX, respectively. In addition, the appearance of new strong and broad peaks at 3433–3427 cm^−1^ and medium peaks at 1658–1650 cm^−1^ are due to N–H stretching and N–H bending in AAm, respectively. This confirms the complete removal of FLX molecules from MIP and good preparation of MIP, which can be used in the sensing part of an applied sensor.

**Figure 3:**
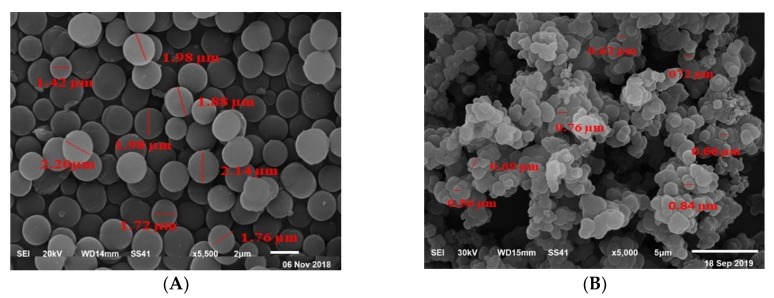
Scanning electron microscope (SEM) images of (**A**) NIP beads and (**B**) washed MIP beads.

As shown in Figure 3, the morphologies of MIP and NIP surfaces were examined by using a scanning electron microscope (SEM). Figure 3A shows the high uniformity of spherical beads of non-imprinted polymer (NIP) with mean diameter of about 1.8 µm. On the other hand, the morphology of washed MIP is showed in Figure 3B as irregular nano-beads with a mean diameter around 0.7 µm. Various morphologies of MIP and NIP confirm the investigation of the imprinting process that ensures efficient MIP as an adequate ionophore in the proposed sensor.

**Figure 4:**
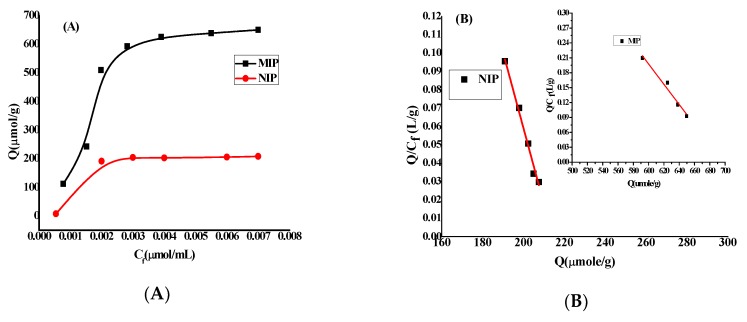
(**A**) Binding isotherm and (**B**) Scatchard plot for MIP and NIP.

The affinities and adsorption capacities of MIP and NIP can be studied by the binding experiment that shows two isotherms in Figure 4A. There are clear differences between MIP and NIP in adsorption capacities for the drug, which reveal that MIP has higher affinity sites than NIP. These sites refer to cavities of the leaved drug molecules after washing beside the functional groups on the surface. The adsorption capacities of polymers increased with increase in FLX concentration. The free FLX concentrations were determined by spectrophotometric technique at *λ_max_* = 226 nm. The binding capacity of MIP and NIP were calculated according to the following equation:

*Q* = *µmol* (*FLX_bound_*)/[*m* (*NIP/MIP*)] = (*C_i_* − *C_f_*) *Vs* × 1000/[*m* (*NIP/MIP*)] (1)

where *Q* is the binding capacity of NIP or MIP (μmol/g), *C_i_*, *C_f_*, *V_s_* and m (NIP/MIP) are the initial FLX concentration (μmol/mL), the final FLX concentration (μmol/mL), the volume of the tested solution (mL) and the mass of dried polymer (g), respectively.

Furthermore, a Scatchard analysis model as shown in Figure 4B was evaluated to give binding capacity and dissociation constant values at binding sites of NIP and MIP according to following equation:

*Q/C_f_* = (*Q_max_* − *Q*)/*K_d_* (2)

where *Q* is the binding capacity, *C_f_*, *Q_max_* and *K_d_* are the free analytical concentration at equilibrium (μmol/mL), the maximum apparent binding capacity and the dissociation constant at binding sites, respectively.

The results of *K_d_* and *Q_max_* values are 500.00 µmol/L and 709.50 µmol/g, respectively for MIP and 250.0 µmol/L and 221.3 µmol/g, respectively for NIP. These values reflect the higher affinities of binding sites of MIP than NIP, which is attributed to formation of specific and selective cavities of MIP through the polymerization steps and after washing the polymer beside the presence of functional binding groups in MIP and NIP. Also, one regression line of two polymers is shown in the Scatchard analysis model that refers to the uniformity and homogeneity of the binding sites of NIP and MIP.

The presence of the template during the polymerization can be measured with the imprinting factor (IF) as follows:

*IF* = [Q*_max_* (*MIP*)]/[*Q_max_* (*NIP*)] (3)

where *Q_max_* (MIP) and *Q_max_* (NIP) are maximum binding capacities of the imprinted and the non-imprinted polymers, respectively. The imprinting factor (IF) is calculated to be 3.2.

### 3.2. Sensor Analytical Features 

FLX is represented as one of the physiologically active amines category that is introduced from pharmaceutical products, which contains an amine functional group [37]. The sensitivity and selectivity of analytes in ISEs can be controlled by introducing the carrier capable of selectively binding of the drug in the membrane by changing the nature of the ion exchanger (additive) or by changing the nature of the plasticizer. The presence of the plasticizer that contains polar or polarizable groups acts as a solvent—mediator of PVC and ionophore, and prevents its exudation from the polymeric matrix to the solution. Therefore, the three different membranes can be prepared to contain MIP as ionophore or carrier with TPB^−^ as an additive and PVC plasticized in DOP, *o-*NPOE or DOS, separately.

For studying the electrochemical behavior and analytical features of an FLX-MIP sensor, three previous membranes were prepared as cocktails in THF and were spotted individually by 10 µL on the on-sensing orifices of the screen-printed electrode, which was modified with 10 µL of MWCNT as a solid contact substance. 

**Figure 5:**
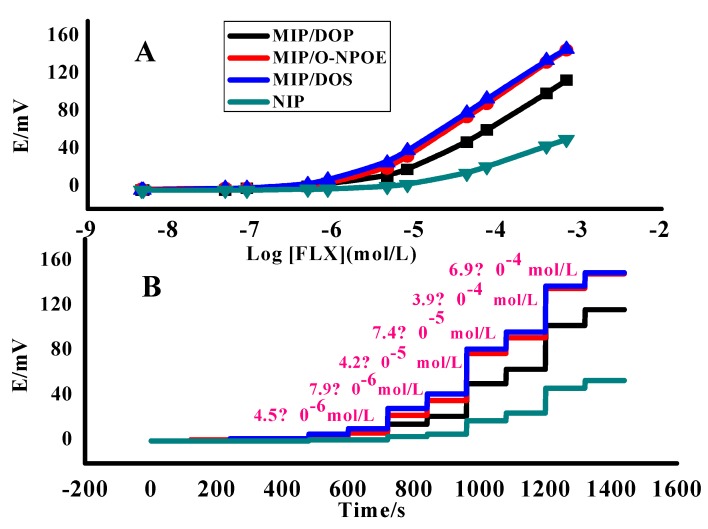
**(A**) Calibration plots and (**B**) time response of non-imprinted polymer (NIP) and the proposed sensors in acetate buffer (0.01 M) pH = 4.5 with multi-walled carbon nanotube (MWCNT) as a transducer.

The influences of the polarity and chemical nature of each plasticizer on the potentiometric response of the proposed sensor were investigated. In order for the plasticizer to be adequate for using in the polymeric liquid membranes in ISEs it must have high molecular weight high lipophilicity, low vapor pressure and high capacity of dissolving the substrate and other components that are found in the polymeric membrane. Additionally, the most important parameters that may affect its behavior are dielectric constant (*ε*_o_) and its viscosity [37]. By comparing the results as can be seen in Table 1, the best cationic slopes response, which is near-Nernestian, is for the electrodes of MIP-FLX membranes; their responses are 57.1 ± 0.3 and 58.9 ± 0.2 mV/decade with detection limits 4.9 × 10^−6^ and 2.1 × 10^−6^ mol/L, plasticized with DOP (*ε*o = 5.1) and *o-*NPOE (*ε*o = 24), respectively. But the best detection limit can be investigated with the membrane plasticized with DOS (εo = 4.6) is 1.4 × 10^−6^ mol/L with near-Nernestian slope (56.0 ± 0.6 mV/decade) (Figure 5).

Although the dielectric constant (*ε*o) of *o-*NPOE is the highest value, its nature can’t affect results purely based on the electrode performance comparing with the nature of the drug that has primary amine active sides and adding the tetraphenyl borate (TPB) as ion-exchanger or additive in the membrane. Therefore, the results confirm that the dielectric constant of the plasticizers, used as solvent-mediators, does not effect in a significant manner the potentiometric detection limit of the membrane in the case of an FLX drug [38].

**Figure 6:**
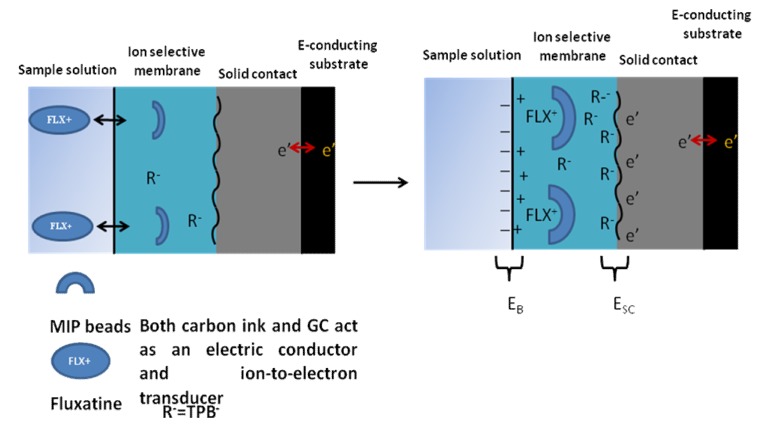
Proposed mechanism for potential development.

Remarkably, the effect of the presence of MIP, NIP and TPB individually in the membranes also can be investigated. Table 1 shows that the sub-Nernestian slope of the sensor of NIP (29.8 ± 1.3 mV/decade) and its detection limit (1.2 × 10^−5^ mol/L) confirm the influence of the imprinting factor of MIP that has higher affinity and sensitivity for FLX molecules with near-Nernestian slope (48.8 ± 0.5 mV/decade) and lower detection limit (8.5 × 10^−6^ mol/L). In the case of the presence of TPB without MIP in the membrane shows the near-Nernestian slope (50.9 ± 0.3 mV/decade) and low detection limit (7.1 × 10^−6^ mol/L) due to its ion-exchange nature. However, it is clear that the presence of both MIP and TPB in the membrane enhances the potentiometric features of the proposed sensor and increases its sensitivity with near-Nernestian slope (57.1 ± 0.3 mV/decade) for detection limit (4.9 × 10^−6^ mol/L). Figure 6 represents a proposed mechanism for the potential development across the membrane.

**Figure 7:**
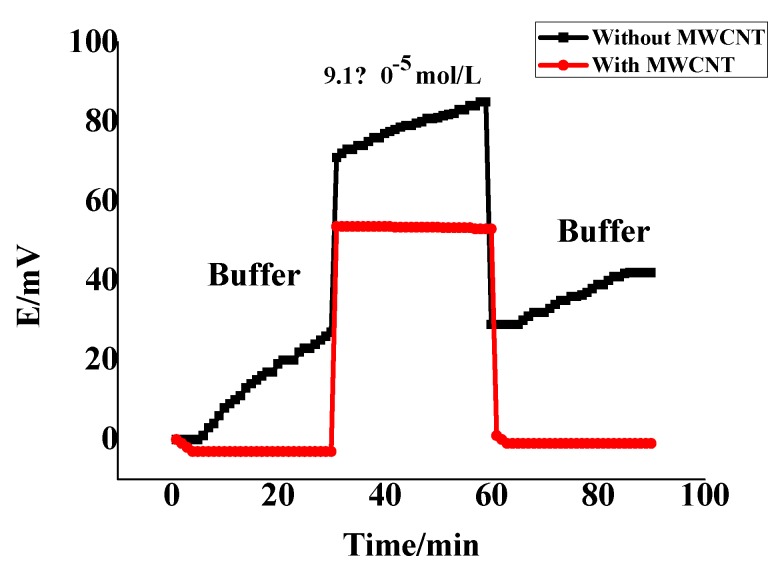
Water-layer tests for the FLX-ISE with and without MWCNT as the solid contact.

As previously shown in Figure 5, the dynamic response of the proposed sensors was examined over the concentration range 1.0 × 10^−7^–1.0 × 10^−2^ mol/L of FLX solution. The results exhibited by response values taken every 3 s over 2 min for each half mL of each concentration show a fast and stable potentiometric response (<10 s). This refers to the high performance of the proposed sensor in the FLX determination. Note the water layer formation test and the comparison between the presence of the MWCNT layer and its absence are shown in Figure 7. MWCNT lipophilicity was tested by immersing the applied sensor, in its two cases of the presence and absence of MWCNT, in the buffer of pH 4.5 for 30 min; then the solution was alternated to be 9.1 × 10^−5^ mol/L of FLX for 30 min and finally was replaced with a buffer for 30 min. As shown in Figure 7, the presence of MWCNT offered the potential stability of the applied sensor over the measuring time and decreased the observed potential drift that exhibited from its absence. This remarkable result confirms the lipophilicity of MWCNT and its role in the decrease of water layer formation that could be the reason for the potential drift and the enhancement of the potential stability of the applied sensor.

### 3.3. Method Validation

The U.S. Food and Drug Administration (FDA) has announced recommendations to support applicants in providing analytical methods, verification data, and samples. These recommendations help to validate the analytical procedures and controls documentation [39]. To ensure the reliability, reproducibility and consistency of the analytical data sets of the proposed sensor of FLX-MIP with its new features, three batches (5 replicates each) of a standard solution of FLX were used to exhibit the linear range, detection limit, precision (standard deviation) and accuracy (trueness), through response stability, method robustness and selectivity.

#### 3.3.1. Detection Limit and Method Linearity

The reliability of the detection limit data was determined by the cross point method according to IUPAC Recommendations [40]. The practical lower limit of detection (LOD) was taken as the concentration of FLX at the point of intersection of the extrapolated linear midrange and final low concentration level segments of the calibration plot as shown in Figure 5a. The exhibited data of LOD are recorded as 4.9 × 10^−6^ (1.7 µg/mL), 2.1 × 10^−6^ (0.7 µg/mL) and 1.4 × 10^−6^ mol/L (0.5 µg/mL) of sensors 1, 2 and 3, respectively. The linear range of each calibration graph was 1.0 × 10^−2^–6.3 × 10^−6^, 1.0 × 10^−2^–5.5 × 10^−6^ and 1.0 × 10^−2^–4.7 × 10^−6^ mol/L of sensors 1, 2 and 3, respectively.

#### 3.3.2. Method for Accuracy and Precision

Accuracy can also be reported as trueness or percent error (closeness of the agreement between the result of a measurement and a true value) that is calculated from Equation (4). The reproducibility of a set of measurements is concluded as precision or standard deviation (RSD), which can be shown in Equation (5).

% *error* = (*Actual value* − *expected value*)/*expected value* × 100 (4)

*S* = √[∑(*xi* − *x*’)^2^/(*n* − 1)] (5)

Alternatively, the standard deviation can be expressed as the percent relative standard deviation, RSD%:

*RSD*% = *S/X*’ × 100 (6)

where x, x_i_ and n are the mean result, one of the n deferent results and number of measurements, respectively. 

The precision of this method was examined by using six replicate measurements of 5 µg/mL of FLX solution. The exhibited results of relative standard deviation were 1.7%, 1.6% and 0.6% of sensors 1, 2 and 3, respectively.

#### 3.3.3. Method Ruggedness (Robustness)

**Figure 8:**
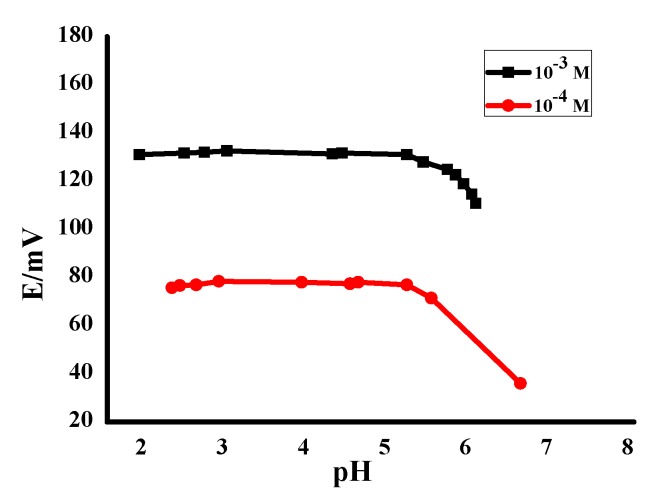
The effect of pH on the potentiometric response of the applied sensor.

The pH dependence of the proposed sensor was examined over the pH range of 2–8. The potential readings were recorded for two solutions of FLX (10^−3^ and 10^−4^ mol/L) at various pHs by using HCl and/or NaOH solutions for adjustments. Figure 8 shows the behavior of the proposed sensor in the different pH values; it shows the wide stability over the pH range from 2 to 5.2, which was used as a working range. However, in the range higher than pH 5.2 a significant decrease in pH was detected. This decrease can be exhibited from the increasing pH and increasing OH^−^ species in the tested solution that caused the FLX base precipitation. A solution of acetate buffer (10 mmol/L) at pH 4.5 was chosen to be a working pH for all potentiometric measurements in this study. 

#### 3.3.4. Sensors’ Selectivity

The potentiometric selectivity of the proposed sensor was investigated by using the modified separate solution method (MSSM) [26]. In addition, the effect of different plasticizers on the selectivity coefficient (*log K ^pot^_FLX, J_*) was tested by using different membranes containing DOP, *o-*NPOE or DOS as solvent-mediators in the proposed sensor. There were various interfering cationic species such as inorganic species (Na^+^, K^+^, Mg^2+^, Ca^2+^ and Ba^2+^), amino acids (alanine and arginine), sugars (glucose and lactose), and organic molecules such as caffeine, with the co-administered drug FLX (Sildenafil) were tested. Some of the previous interferences can be found in the dosage forms of FLX or in the biological fluids. As shown in Table 2, the results reflect no significant interference of these species with FLX determination. However, the best values of log *K ^pot^_FLX, J_* are in the case of using DOS as a plasticizer in the proposed sensor.

### 3.4. Potential Stability

**Figure 9:**
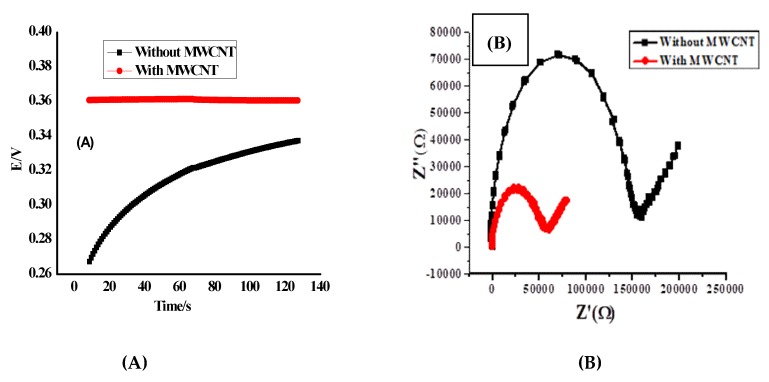
(**A**) Chronopotentiometry and (**B**) impedance plot for FLX/MIP-ISEs, with and without MWCNT as a solid contact material. Abbreviations: ion-selective electrodes (ISEs).

The short-term potential stability of the all-solid-state was evaluated by applying the constant-current chronopotentiometry (CP) technique. This technique has been developed by Bobacka’s group [39]. All the measurements were applied in a solution of 10^−3^ mol/L of FLX in acetate buffer (10 mmol/L) of pH 4.5 at room temperature 25 °C ± 1 °C by using a one-compartment three-electrode cell using (NOVA 2.0 software; Metrohm Auto lap B.V. Utrecht, The Netherlands) attached with a Pt auxiliary electrode and a reference electrode (Ag/AgCl/KCl (3 mol/L). The applied current is ± 1 nA for the 60 s. As shown in Figure 9A, the comparison between the potential stability of the applied sensor in the presence and the absence of MWCNT as a solid contact material and transducer was investigated. The bulk resistance (R_b_) that exhibited from the membrane components that equal 0.35 MΩ was affected by the presence of MWCNT to be 0.19 MΩ. Exhibited capacitance (C_L_) values incredibly increased from 3.7 µF to 160.0 µF in the presence of MWCNT as a good transducer. The potential drift (∆E/∆t) in cases of absence and presence of MWCNT was recorded as 270.0 and 6.3 µV/s, respectively. The previous results reflect the high performance of the proposed sensor in the presence of MWCNT, which increases the conductance and decreases the resistance and the potential drift over the time of the measurements.

### 3.5. Electrochemical Impedance Spectrometry (EIS)

The electrochemical impedance (EIS) measurements were tested by using the same device that was used in CP measurements. The range of frequencies were applied between 100 kHz to 0.1 Hz using a sinusoidal excitation signal with an excitation amplitude of 0.01 V of open-circuit potential [41]. Figure 9B show the Nyquist plots on the equivalent circuit models. The two plots of the proposed sensor in the presence and absence of MWCNT reflect resistance of the membrane value equal to 0.06 and 0.16 MΩ, respectively. The double layer capacitances (C_dl_) were estimated from the low-frequency branch (semicircle), which were valued as 86.9 and 42.4 µF in the case of the presence and absence of MWCNT, respectively. These values exhibited due to the nano-structured features of MWCNTs, which generate a large double-layer capacitance and increase the potential stability. The geometric capacitances (C_g_) were estimated at high frequencies part that valued as 1.5 and 0.6 nF for presence and absence of MWCNT, respectively. The whole results exhibited from CP and EIS reflect the enhancement of the performance of the applied sensor in the presence of MWCNT, which helps to increase the capacitance and decrease the potential drift duration of the measurements.

### 3.6. Analytical Applications

The exhibited data from FLX determination in different dosage forms by using the proposed sensor are shown in Table 3. Recoveries’ results were ranged between 98.6%–101.1%. The results were compared with the reference method of HPLC from British Pharmacopeia (B.P.) [42]. The *t*-student and *F*-tests were calculated for two methods and showed no significant difference between the two methods, which confirms that the proposed method is an applicable and efficient method of FLX determination in their different matrices.

## 4. Conclusions

A facile and validated potentiometric method for fluoxetine (FLX) monitoring based on solid-contact ISEs was presented. MIP for FLX was prepared, dispersed in plasticized PVC membrane and drop-casted on the orifice of the screen-printed electrode. A multi-walled carbon nanotube (MWCNT) was used as nanomaterial for the ion-to-electron transduction process. The presented sensor revealed a stable response with a Nernstian slope of 58.9 ± 0.2 mV/decade and a detection limit of 2.1 × 10^−6^ mol/L in 10 mmol/L acetate buffer of pH 4.5. The effect of plasticizers on the potentiometric response and selectivity behavior was studied. The presented miniaturized ISEs revealed enhanced repeatability, reproducibility and stability. Validation of the assay method with the proposed ISE sensors, by measuring the lower detection limit, range, accuracy, precision, repeatability and between-day-variability, reveals good performance characteristics confirming applicability for continuous determination of FLX in pharmaceutical formulations.

## Figures and Tables

**Table 1:** The potentiometric response of the fluoxetine (FLX) membranes sensor.

No	MIP, mg	NIP, mg	TPB, mg	Plasticizer, mg			PVC, mg	* Slope, (mV/Decade)	Detection Limit, (mol/L)	r^2^
				DOP	o-NPOE	DOS				
1	12.00	-	2.00	118.00	-	-	68.00	57.1 ± 0.3	4.9 × 10^−6^	0.999
2	12.00	-	2.00	-	118.00	-	68.00	58.9 ± 0.2	2.1 × 10^−6^	0.999
3	12.00	-	2.00	-	-	118.00	68.00	56.0 ± 0.6	1.4 × 10^−6^	0.999
4	12.00	-	-	118.00	-	-	68.00	48.8 ± 0.5	8.5 ×10^−6^	0.999
5	-	12.00	-	118.00	-	-	68.00	29.8 ± 1.3	1.2 ×10^−5^	0.999
6	-	-	2.00	118.00	-	-	68.00	50.9 ± 0.3	7.1 × 10^−6^	0.999

* Average of 6 determinations.

**Table 2:** The selectivity coefficients (log K ^pot^_FLX, J_) of the proposed sensor.

Interfering Ion	−log K ^pot^_FLX, J_		
	DOP	*o*-NPOE	DOS
**Na^+^**	5.0	5.0	5.7
**K^+^**	4.7	5.0	5.3
**Mg^2+^**	5.0	4.9	5.4
**Ca^2+^**	5.0	4.9	5.6
**Ba^2+^**	4.7	4.9	5.6
**Alanine**	4.9	4.7	5.6
**Arginine**	7.1	6.1	7.1
**Glucose**	5.0	5.0	5.7
**Lactose**	4.7	5.0	5.7
**Caffeine**	5.0	5.0	5.7
**Sildenafil**	3.2	2.8	3.5

**Table 3:** FLX determination in pharmaceutical preparations using the proposed membrane sensor and reference method.

Pharmaceutical Product and Source	Nominal Content Taken, mg tablet^−1^	Found, mg tablet^−1^				t-Student Test	*F*-Test
		Proposed Method	Mean ^a^ (%) ± SD	Reference Method [42]	Mean ^a^ (%) ± SD		
Prozac (Lilly, France)	**20**	**20.2**	101.0 ± 0.3	20.1	100.8 ± 0.6	0.4	4.4
Philozac (Amoun, Egypt)	20	19.8	99.2 ± 0.6	19.9	99.1 ± 1.7	0.2	8.7
Flutin (Eipico, Egypt)	20	20.3	101.1 ± 0.6	19.8	99.4 ± 0.9	2.3	2.6
Depreban (Amirya, Egypt)	20	19.7	98.6 ± 0.4	19.4	97.2 ± 0.8	1.7	2.9

^a^ Mean of three replicate measurements ± standard deviation (SD). ^b^ *t*-Student and F-test at 95% confidence level values are 4.30, 19.00 respectively.
